# 10-Gingerol Increases Antioxidant Enzymes and Attenuates Lipopolysaccharide-Induced Inflammation by Modulating Adipokines in 3T3-L1 Adipocytes

**DOI:** 10.3390/antiox13091093

**Published:** 2024-09-07

**Authors:** María Elizabeth Preciado-Ortiz, Erika Martínez-López, José Pedraza-Chaverri, Omar Noel Medina-Campos, Roberto Rodríguez-Echevarría, Samantha Desireé Reyes-Pérez, Juan José Rivera-Valdés

**Affiliations:** 1 Doctorado en Ciencias de la Nutrición Traslacional, Departamento de Clínicas de la Reproducción Humana, Crecimiento y Desarrollo Infantil, Centro Universitario de Ciencias de la Salud, Universidad de Guadalajara, Guadalajara 44340, Mexico; elizabeth.nuta@gmail.com; 2 Instituto de Nutrigenética y Nutrigenómica Traslacional, Departamento de Biología Molecular y Genómica, Centro Universitario de Ciencias de la Salud, Universidad de Guadalajara, Guadalajara 44340, Mexico; roberto.rodriguez@academico.udg.mx (R.R.-E.); sade.reyes.perez@gmail.com (S.D.R.-P.); 3 Departamento de Biología, Facultad de Química, Universidad Nacional Autónoma de México, Ciudad de México 04510, Mexico; pedraza@unam.mx (J.P.-C.); omarnoelmedina@gmail.com (O.N.M.-C.); 4 Doctorado en Ciencias en Biología Molecular en Medicina, Departamento de Biología Molecular y Genómica, Centro Universitario de Ciencias de la Salud, Universidad de Guadalajara, Guadalajara 44340, Mexico

**Keywords:** gingerol, adipocyte, ginger, antioxidant, anti-inflammatory, obesity

## Abstract

Background: Obesity increases reactive oxygen species production and alters adipokines levels, resulting in a low-grade chronic inflammation state, which contributes to tissue metabolic dysfunction. 10-gingerol, a phenol present in ginger, has shown potential anti-obesogenic effects in vitro. However, the antioxidant and anti-inflammatory properties of 10-gingerol have not been approached. The aim of this study was to investigate the effects of 10-gingerol on antioxidant enzymes’ expression and adipokine production in 3T3-L1 adipocytes in response to lipopolysaccharide (LPS)-induced inflammation. Methods: 10-gingerol antioxidant capacity was assessed through Oxygen Radical Absorbance Capacity (ORAC) , Ferric Reducing Antioxidant Power (FRAP), and radical scavenging activity of 2,2-diphenyl-2-picrylhydrazyl (DPPH) assays. 3T3-L1 cells were differentiated and stimulated with 100 ng/mL LPSs. Then, 15 µg/mL 10-gingerol was added for 48 h. The mRNA expression and protein abundance of antioxidant enzymes were evaluated by qPCR and Western blot, respectively. Adipokine levels were determined by ELISA. Results: 10-gingerol showed low FRAP and DPPH values but a moderate ORAC value. Moreover, 10-gingerol increased *Gpx1* and *Sod1* but downregulated *Cat* expression. Additionally, 10-gingerol significantly increased CAT and GPx1 levels but not SOD-1. Finally, adiponectin and leptin concentrations were increased while resistin and tumor necrosis factor alpha (TNFα) were decreased by 10-gingerol. Conclusions: 10-gingerol presented antioxidant potential by increasing antioxidant enzymes and attenuated LPS-induced inflammation by modulating adipokines in 3T3-L1 adipocytes.

## 1. Introduction

Obesity represents a worldwide unresolved epidemic with multiple comorbidities associated. This condition has tripled during the last 50 years in the adult population and continues to grow rapidly [1,2]. Obesity is generally derived from a chronic imbalance between calorie intake and energy expenditure, leading to adipose tissue hypertrophy in adults [3]. Nowadays, obesity stands out as the main risk factor for the development of other conditions such as cardiovascular disease, type 2 diabetes, dyslipidemia and cancer as a result of low-grade chronic inflammation [4,5].

Obesity is associated with increased production of reactive oxygen species (ROS) and inflammatory adipokines such as resistin, tumor necrosis factor alpha (TNFα), and interleukin 6 (IL-6), as well as a reduction in anti-inflammatory adipokines (adiponectin) [4,6]. Adipokines are molecules secreted by adipocytes that play an important role in body weight control, lipid metabolism, insulin sensitivity, inflammation, vascular function, and immune regulation [7]; however, their production is altered in obesity due to excess body fat [3,8,9]. Furthermore, the release of free fatty acids and inflammatory cytokines in response to chronic exposure to ROS contributes to the exacerbation of oxidative stress and the development of a state of chronic low-grade inflammation, which creates a vicious cycle [6,10] and contributes to tissue metabolic dysfunction [3,8,9,11].

It has been reported that free radicals and ROS activate the nuclear factor kappa-light-chain enhancer of the activated B cell (NF-κB) pathway, promoting its translocation to the nucleus to further promote the expression of pro-inflammatory mediators like TNFα, IL-6, adhesion molecules, and enzymes like the inducible nitric oxide synthase (iNOS) and cyclooxygenase-2 (COX-2) [12,13]. These reactive species can also induce lipid peroxidation, leading to the formation of reactive lipid peroxidation products such as malondialdehyde (MDA) and 4-hydroxynonenal (4-HNE), which have the potential to further propagate oxidative stress and inflammation by damaging proteins and DNA [14]. In fact, the interplay between oxidative stress and inflammation is pivotal in the development of obesity-related complications due to the direct damage to insulin-sensitive tissues [11,15]. Therefore, the inability of the endogenous antioxidant enzymes to counteract free radicals and ROS in adipose tissue might lead to the onset of insulin resistance, which is in turn a hallmark of metabolic syndrome and type 2 diabetes mellitus [15,16].

Importantly, it is by the action of enzymes like superoxide dismutase (SOD), catalase (CAT), and glutathione peroxidase (GPx) that the neutralization of ROS occurs. In obesity, the expression and activity of these enzymes might be compromised [16]. As for human diet, certain micronutrients and phytophenols are able to scavenge free radicals directly and also enhance the expression of the antioxidant enzymes and other related proteins [17,18]. Thus, an adequate supply of these molecules is vital to counterbalance the exacerbated production of ROS in adipose tissue in the context of obesity [17,18].

Therefore, there is a growing interest for research in bioactive food compounds that enhance the expression of the antioxidant enzymes [17,18], ameliorate pro-inflammatory cytokine production, and normalize adiponectin levels to supply some useful insights in the development of complementary strategies for the prevention and treatment of obesity-related complications [17,19].

In this regard, several plants with health-promoting properties have been under the spotlight for their capacity to support human health given their bioactive compounds. One example of this is ginger (*Zingiber officinale roscoe*), which has garnered attention due to its anti-inflammatory, anti-obesogenic, antiemetic, and antioxidant effects [20,21,22]. The consumption of ginger in the human diet provides a variety of phenolic compounds, including gingerols, as the most abundant, followed by shogaols and paradols [23]. Within the list, gingerols represent the vast majority of phenolic compounds in fresh ginger and include 6-, 4-, 5-, 8-, 10-, and 12-gingerols, and it is thought that they could be potential contributors to its major therapeutic effects [24,25]. 10-gingerol is one of the most abundant gingerols in ginger root, which exhibits higher bioavailability after consumption [26] and has shown beneficial effects on adipocytes [21], although its antioxidant and anti-inflammatory properties have not been studied. Thus, the aim of our study was to investigate the effects of 10-gingerol on antioxidant enzyme expression and adipokine production in 3T3-L1 adipocytes in response to lipopolysaccharide (LPS)-induced inflammation. By elucidating the molecular mechanisms through which 10-gingerol exerts its effects, this research aims to provide insights into its potential as a therapeutic agent for combating obesity-related oxidative stress and inflammation.

## 2. Materials and Methods

### 2.1. 10-Gingerol Preparation

10-gingerol was purchased from Sigma-Aldrich Chemical Co. (Sigma-Aldrich, St. Louis, MO, USA) at a standard concentration of 5 mg/mL and reconstituted in methanol (JT Baker, Xalostoc, Ecatepec, Mexico). A stock solution and dilutions were made in dimethyl sulfoxide (DMSO, Sigma-Aldrich, St. Louis, MO, USA).

### 2.2. Antioxidant and Free Radical Scavenging Activities of 10-Gingerol

#### 2.2.1. Oxygen Radical Absorbance Capacity (ORAC) Assay

This antioxidant determination was made according to the method described by Fernández-Rojas and collaborators [27]. Briefly, Trolox standards (0–10 μM) and diluted 10-gingerol were added to the reaction mixture containing 19 mM 2,2-azobis(2-amidinopropane) dihydrochloride (AAPH) (Sigma-Aldrich, St. Louis, MO, USA) and 23 nM fluorescein (Sigma-Aldrich, St. Louis, MO, USA). Fluorescence was measured for 90 min at excitation and emission wavelengths of 485 and 520 nm, respectively, using the Synergy HT multi-detection microplate reader (Biotek Instruments Inc., Winooski, VT, USA). The ORAC values were calculated using the net area under the decay curves obtained by Gen5 software (version 3.0, Biotek Instruments, Santa Clara, CA, USA) and were expressed as millimoles of Trolox equivalents (TE)/g of the sample.

#### 2.2.2. Ferric Reducing Antioxidant Power (FRAP) 

The antioxidant capacity of a molecule is revealed when it reacts with the colorless ferric tripyridyl triazine (Fe^3+^-TPTZ) complex and forms blue ferrous tripyridyl triazine (Fe^2+^-TPTZ). Briefly, dilutions of 10-gingerol were mixed with FRAP reagent [0.833 mM 2,4,6-Tris(2-pyridyl)-s-triazine (TPTZ) (Sigma-Aldrich, St. Louis, MO, USA), 1.66 mM FeCl_3_ (Sigma-Aldrich, St. Louis, MO, USA), in 250 mM acetate buffer, pH 3.6]. Fifteen minutes later, absorbance at 593 nm was assessed using a Synergy HT multi-mode microplate reader (Biotek Instruments, Winooski, VT, USA). Results were expressed as millimoles of TE (Sigma-Aldrich, St. Louis, MO, USA)/g of the sample. 

#### 2.2.3. DPPH Radical Scavenging Assay

In order to determine the radical scavenging activity of 2,2-diphenyl-2-picrylhydrazyl (DPPH) and radical scavenging of 10-gingerol, the method reported by Ignacio-Mejía et al. [28] was used. Trolox was used as a positive control reagent. Briefly, 20 μL of 10-gingerol, Trolox (0.03–0.5 mM), or water, were mixed with 34 μL of 2 mM DPPH (Sigma-Aldrich, St. Louis, MO, USA). After 2 min, 216 μL of ethanol (JT Baker, Xalostoc, Ecatepec, Mexico) was added to the mixture, which was then incubated in the dark for an additional 2 min at room temperature. Optical density at 517 nm was measured using a Synergy HT multimode microplate reader (Biotek Instruments, Winooski, VT, USA). The percentage of DPPH radical scavenging was calculated as follows:

% DPPH scavenging = [(Absorbance control − Absorbance sample)/Absorbance sample) × 100]

Subsequently, a linear plot of % inhibition versus concentration was analyzed. The equation used is [29]

*y* = a + b(*x*),

where *x* represents the concentration of the substance being measured, and *y* denotes the % inhibition. The IC50 value was determined as the concentration (*x*) at which the % inhibition (*y*) is 50% [29]. 

### 2.3. Cell Culture 

#### 2.3.1. Culture and Differentiation of 3T3-L1 Cells

The 3T3-L1 cells (ATCC^®^ CL-173™) were generously donated by Dra. Trinidad Garcia-Iglesias of the University Center of Health Sciences, University of Guadalajara (Guadalajara, Mexico).

3T3-L1 cells were maintained at 37 °C with 5% CO_2_ in a humidified incubator. Cells were seeded in Petri dishes at a density of 1 × 10^5^ cells/dish with Dulbecco’s Modified Eagle Medium (DMEM; Sigma-Aldrich; Merck KGaA, St. Louis, MO, USA) supplemented with 10% calf bovine serum (CBS; Cytiva; HyClone, Logan, UT, USA) and 1% antibiotics (100 U/mL penicillin and 100 μg/mL streptomycin (Gibco; Thermo Fisher Scientific, Inc., Waltham, MA, USA)). The medium was replaced every 2–3 days until the cells reached 100% confluence. Differentiation was induced following the manufacturer’s protocol (3T3-L1 Differentiation Kit; Sigma-Aldrich; Merck KGaA, St. Louis, MO, USA). Upon reaching confluence (day 0), the growth medium was replaced with differentiation medium containing DMEM/F12 (Gibco; Thermo Fisher Scientific Inc., Waltham, MA, USA) supplemented with 10% fetal bovine serum (FBS; Gibco; Thermo Fisher Scientific Inc., Waltham, MA, USA), 1% antibiotics, and an adipogenic cocktail including 500 μM 3-Isobutyl-1-methylxanthine, 1 μM dexamethasone, 1.5 μg/mL insulin, and 1 μM rosiglitazone (3T3-L1 Differentiation Kit; Sigma-Aldrich; Merck KGaA, St. Louis, MO, USA) for 72 h. Subsequently, the medium was switched to a maintenance medium comprising DMEM/F12 with 10% FBS, 1% antibiotics, and 5 μg/mL insulin, which was refreshed every 2 days for a total of 8 days of differentiation. 

#### 2.3.2. 3T3-L1 Cell Treatments

**Figure 1:**
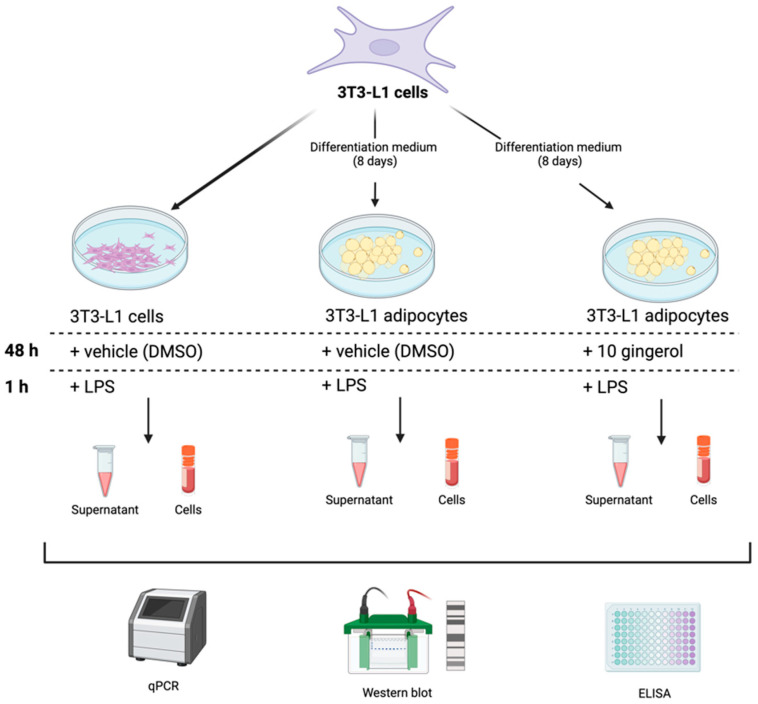
In vitro study design to determine 10-gingerol effects in 3T3-L1 adipocytes in response to lipopolysaccharide-induced inflammation.

Three study groups were formed: the first group consisted of undifferentiated 3T3-L1 cells, and the second group consisted of 3T3-L1 cells differentiated into adipocytes over 8 days (Adipocytes group). Both groups were stimulated with 0.5% DMSO as a vehicle. The last group were 3T3-L1 cells differentiated into adipocytes (8 days of differentiation) treated for 48 h with a medium containing 15 µg/mL of 10-gingerol and 0.5% of DMSO (10-G group). The dosage was determined using a cytotoxicity curve (Table S1), and the experimental conditions were based on a previous study [21]. All groups were incubated with medium with LPS (100 ng/mL) for 1 h before being harvested to promote an inflammatory environment [30]. Supernatants from study groups were collected in 1.5 mL microtubes and stored at −80 °C until use. Finally, 3T3-L1 preadipocytes and adipocytes were cryopreserved at −80 °C for experimental analysis (Figure 1). 

### 2.4. Antioxidant Enzyme Expression

#### 2.4.1. mRNA Expression by Quantitative Polymerase Chain Reaction (qPCR)

Total ribonucleic acid (RNA) was isolated from all samples using the RNeasy Mini Kit following the manufacturer’s protocol (Qiagen GmbH, Hilden, Germany). Subsequently, cDNA was synthesized from 1 μg of total RNA using M-MLV Reverse Transcriptase (Thermo Fisher Scientific Inc., Waltham, MA, USA) according to the manufacturer’s instructions. Gene expression analysis was performed by quantitative PCR using a LightCycler 96 Thermocycler (Roche Diagnostics, Indianapolis, IN, USA) with OneTaq^®^ Hot Start Master Mix (NEB-R, Ipswich, MA, USA) and TaqMan^®^ probes (Thermo Fisher Scientific Inc., Waltham, MA, USA) as follows: 18S ribosomal RNA (rRNA) (*Rn18s*; cat. no. 4331182; Assay ID: Mm03928990_g1), catalase (*Cat*, cat. no. 4331182; Assay ID: Mm00437992_m1), superoxide dismutase 1 (*Sod1,* cat. no. 4331182; Assay ID: Mm01344233_g1), and glutathione peroxidase 1 (*Gpx1*, cat. no. 4331182; Assay ID: Mm04207457_g1). Thermocycling was carried out for 300 s, followed by 30 cycles of denaturation at 95 °C for 20 s, amplification at 60 °C for 60 s, and final extension at 68 °C for 300 s. Relative gene expression was determined based on the 2^−ΔΔCt^ method [31] and normalized against the mRNA expression of *Rn18s*. All quantifications were independently performed four times. 

#### 2.4.2. Western Blot Analysis

The precise methodology for cell lysis and protein isolation has been extensively explained before [21]. First, 20 μg of protein was separated with 10% sodium dodecyl sulfate-polyacrylamide gel electrophoresis (SDS-PAGE) and transferred to polyvinylidene (PVDF) membranes (Bio-Rad Laboratories, Inc., Hercules, CA, USA). Later, membranes were blocked for 2 h at room temperature with 5% nonfat dry milk, followed by an overnight incubation at 4 °C with primary antibodies against CAT (1:1000), SOD-1 (1:1000), and GPx-1/2 (1:1000). All antibodies were purchased from Santa Cruz Biotechnology, Inc, Dallas, TX, USA. Subsequently, the membranes were exposed to a horseradish peroxidase (HRP)-conjugated goat anti-mouse secondary antibody (1:50,000; LI-COR Biosciences, Lincoln, NE, USA) for 90 min at room temperature. Protein bands were visualized using the Immobilon Western Chemiluminescent HRP substrate kit (Merck KGaA, Darmstadt, Germany). Detection and quantification of protein signals were performed with the C-Digit Blot Scanner (LI-COR Biosciences, Lincoln, NE, USA) and analyzed using Image Studio software (version 5.5.4, LI-COR Biosciences, Lincoln, NE, USA) for C-Digit Processing. Band density quantifications were normalized to β-actin (1:2000; Thermo Fisher Scientific Inc., Waltham, MA, USA).

### 2.5. Adipokine and Pro-Inflammatory Cytokine Concentrations

The concentrations of adiponectin, leptin, resistin, TNFα, and IL-6 in the culture media were determined by sandwich enzyme-linked immunosorbent assays (ELISAs) (Thermo Fisher Scientific Inc., Waltham, MA, USA). All the protocols were performed according to the manufacturer’s guidelines. Sample concentrations were determined using a microplate reader (MultiScan GO; Thermo Fisher Scientific, Inc.). Experiments were performed in triplicate. 

### 2.6. Statistical Analysis

Data were expressed as mean ± standard error of the mean (SEM). The Shapiro–Wilk normality test was used to determine the normal distribution of quantitative variables. Data were analyzed by one-way analysis of variance (ANOVA) followed by Tukey’s post hoc test to determine differences among group means. A value of *p* < 0.05 was established as a significant difference. Data were analyzed with Prism version 10 (GraphPad Software, San Diego, CA, USA).

## 3. Results

### 3.1. 10-Gingerol Showed Low Antioxidant and Free Radical Scavenging Activity

**Figure 2:**
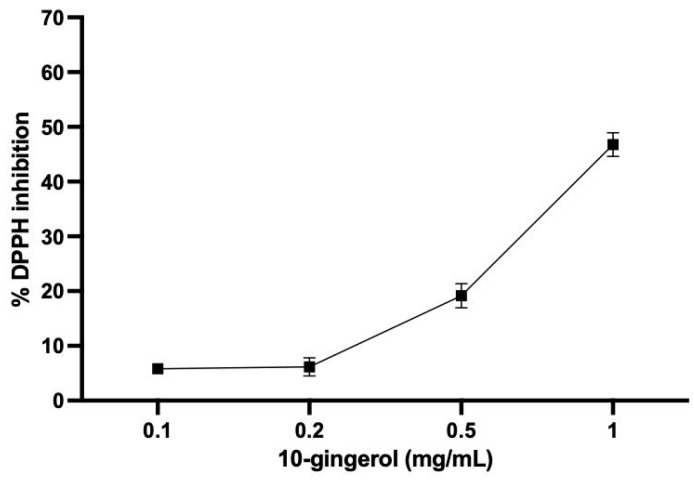
Percentage of DPPH radical scavenging activity of 10-gingerol. Values plotted represent mean ± SEM. The assays were performed in triplicate. DPPH: 2,2-diphenyl-1-picrylhydrazyl radical.

To expand the background of 10-gingerol and have a first approach to its antioxidant capacity, the values of ORAC and FRAP were determined, and the DPPH radical inhibition was estimated. In our results, 10-gingerol presented an ORAC value of 0.12 ± 0.01 mM Trolox equivalents/g and an FRAP value of 0.44 ± 0.05 mM TE/g. In addition, 10-gingerol showed 46.7% DPPH inhibition at 1 mg/mL (Figure 2), and an IC50 value of 1.09 mg/mL.

### 3.2. 10-Gingerol Modulates the Expression of Antioxidant Enzymes in 3T3-L1 Adipocytes Exposed to LPS

**Figure 3:**
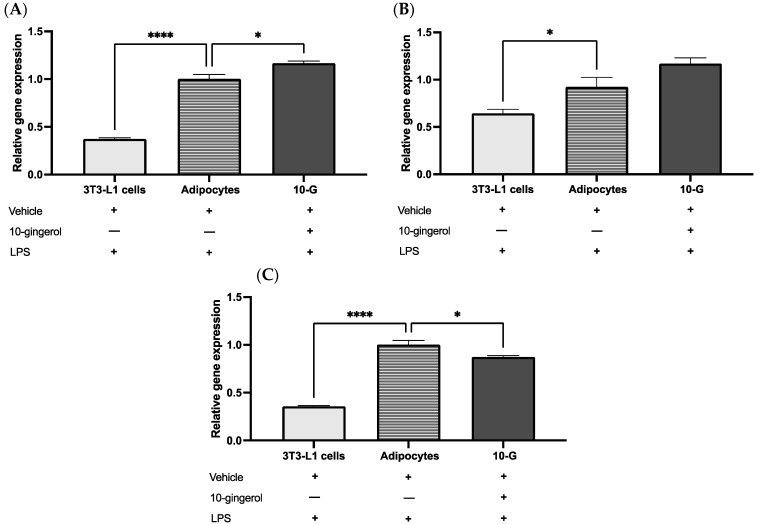
Effect of 10-gingerol on mRNA levels (**A**) *Glutathione peroxidase 1* (*Gpx1)*, (**B**) *Superoxide dismutase 1* (*Sod1*), and (**C**) *Catalase* (*Cat*). The relative gene expression of each sample was normalized to *Rn18s*. Values plotted represent mean ± SEM. The assays were performed in quadruplicate. Differences were analyzed using one-way ANOVA and Tukey’s post hoc. * *p* < 0.05, **** *p* < 0.0001. LPS, lipopolysaccharide.

The expression of genes encoded to antioxidant enzymes in 3T3-L1 adipocytes in response to LPS-induced inflammation and treated with 10-gingerol or a vehicle was assessed. The 10-G group showed significantly increased expression of *Gpx1* (*p* < 0.05) and a tendency to increase *Sod1* expression (*p* = 0.056) compared with the adipocyte group (Figure 3A,B). Nevertheless, 10-gingerol downregulates *Cat* levels compared to the adipocyte group (*p* < 0.05) (Figure 3C).

**Figure 4:**
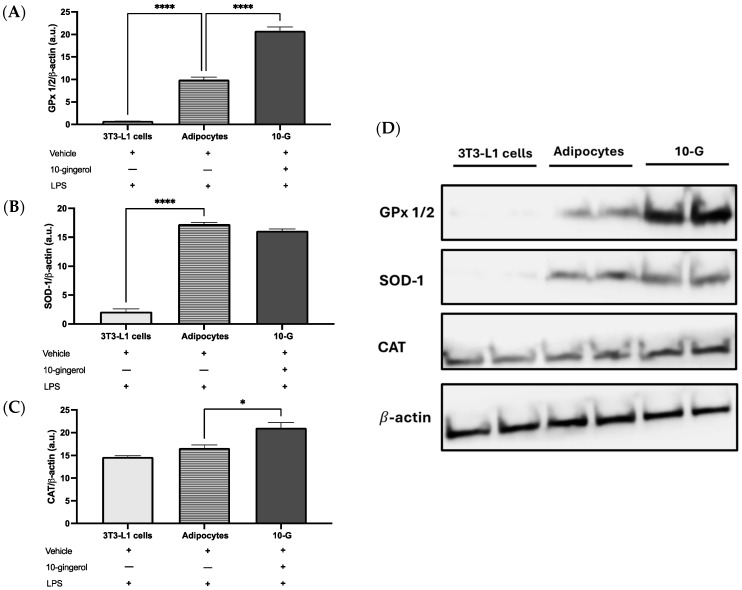
10-gingerol increases antioxidant enzyme abundance in 3T3-L1 adipocytes. (**A**) Glutathione peroxidase 1/2 (GPx 1/2), (**B**) superoxide dismutase 1 (SOD-1), (**C**) catalase (CAT), and (**D**) representative Western blot. β-actin was used as a loading control and was run in a different gel. The assays were performed in duplicate. Values plotted represent mean ± SEM. Differences were analyzed using one-way ANOVA and Tukey’s post hoc. * *p* < 0.05, **** *p* < 0.0001. LPS, lipopolysaccharide.

Regarding the protein levels of antioxidant enzymes, the 10-G group presented significantly increased GPx 1/2 and CAT levels than the adipocyte group (Figure 4A,C,D). No significant differences were observed in SOD-1 levels among the 10-G group and adipocyte group (Figure 4B,D).

### 3.3. 10-G Ameliorates LPS-Induced Inflammation by Regulating Adipocytokines in 3T3-L1 Adipocytes

**Figure 5:**
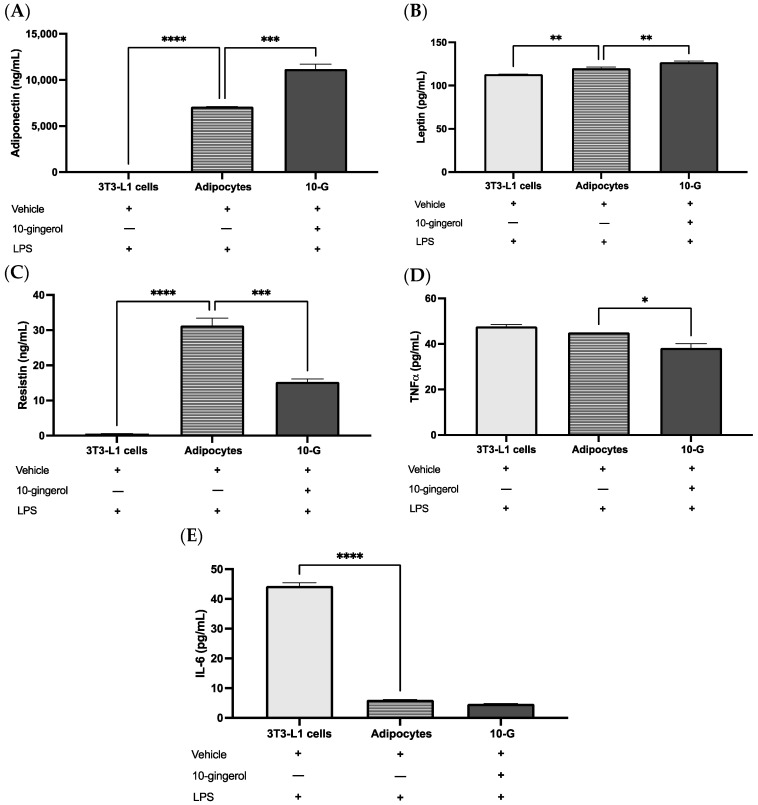
10-gingerol attenuates LPS-induced inflammation by modulating adipokines in 3T3-L1 adipocytes. 10-gingerol increases (**A**) adiponectin and (**B**) leptin concentrations, reduces (**C**) resistin and (**D**) TNFα concentrations, but does not affect (**E**) IL-6 levels in a state of LPS-induced inflammation in 3T3-L1 adipocytes. Values plotted represent mean ± SEM. The assays were performed in triplicate. Differences were analyzed using one-way ANOVA and Tukey’s post hoc. * *p* < 0.05, ** *p* < 0.01, *** *p* < 0.001, **** *p* < 0.0001. LPS, lipopolysaccharide.

To determine the levels of adipokines in the supernatant, after 10-gingerol treatment and acute stimulation with LPS, the ELISA technique was used. Stimulation with 10-gingerol increased adiponectin concentration (*p* < 0.0001) and decreased resistin (*p* < 0.0001) and TNFα (*p* = 0.014) levels compared with the adipocyte group. Also, mature adipocytes stimulated with 10-gingerol showed higher leptin levels than the adipocyte group (*p* = 0.008). No significant differences were observed in IL-6 levels among the 10-G and adipocytes groups (*p* = 0.29), but the 3T3-L1 cells demonstrated higher IL-6 levels compared with the other groups (*p* < 0.0001) (Figure 5).

## 4. Discussion

The search for bioactive compounds with antioxidant and anti-inflammatory properties in plants has aroused scientific interest because these effects can be used to combat oxidative stress and inflammation processes typical of chronic degenerative diseases. In the present study, 10-G demonstrated antioxidant capacity by stimulating the production of the main antioxidant enzymes, as well as great potential to modulate LPS-induced inflammation by regulating adipokines in 3T3-L1 adipocytes.

Ginger is known for containing a large number of compounds with antioxidant activity, such as terpenoids, alkaloids, and polyphenols; the latter group includes gingerols. Some studies have demonstrated the antioxidant capacity of ginger root, stem, and leaves through assays like ORAC, FRAP, or DPPH [32,33,34]; however, there are few studies that have isolated and specifically evaluated the antioxidant capacity of individual ginger compounds such as gingerols. 

ORAC and FRAP are the most well-known methods for detecting antioxidant capacity. In our results, 10-gingerol reached ORAC values similar to those reported with fresh ginger extracts [32,33], while compared to other spices such as turmeric, our results were lower, but higher than black pepper [33]. However, the ORAC value for 10-gingerol was notably lower compared to the antioxidant controls reported in another study, such as ascorbic acid with 5330 mM TE/g and gallic acid with 7880 mM TE/g [35].

On the other hand, 10-gingerol exhibited a low ferric reducing antioxidant power, with a FRAP value of 0.44 mM TE/g. However, the difference in the units reported for FRAP values made comparison difficult. A 2020 study showed a FRAP value of 30.92 mg Trolox/g for ginger root [36]; while a value of 10 μM FeSO_4_/μg was obtained for a ginger plant extract [32]. In addition, one study reported FRAP values ranging from 368.2 to 767.2 μM Fe (II)/g dry weight in different varieties of ginger, with the root showing the highest FRAP value compared to the leaves and stem of the plant, while the positive control, vitamin C, reached 3107.28 µmol Fe (II)/g dry weight [34]. 

DPPH is another method used to assess antioxidant capacity, through antioxidants’ ability to donate hydrogen [37]. Regarding the DPPH radical inhibition capacity, 10-gingerol exhibited an IC50 value of 1.09 mg/mL, which is considered weak [29]. Higher capacity has been reported with 40 ug/mL methanolic extracts of ginger, where a DPPH inhibition percentage of 51.1 to 56.3% has been observed for ginger leaf extract and 51.4 to 58.2% for ginger root extract [34]. Despite our similar ORAC but lower FRAP and DPPH values than those shown in other studies, it should be considered that our results pertain to 10-gingerol, a single bioactive compound and not a mixture of compounds as in the previous studies.

Antioxidants neutralize free radicals and prevent oxidative stress directly or by promoting the production and activity of antioxidant enzymes. Antioxidant enzymes maintain redox balance and protect against oxidative damage. It has been reported that ginger initiates the replenishment of antioxidant enzymes [24,38], which is in agreement with our results. 

A previous study showed that the gene expression levels of *Hmox1*, which encodes heme oxygenase 1 (HO-1), a powerful antioxidant that is crucial in reducing ROS, were significantly upregulated by a ginger extract in 3T3-L1 adipocytes [39]. In the present research, we evaluated both the mRNA and protein levels of three of the most representative enzymes of the endogenous antioxidant system: CAT, SOD-1, and GPx 1/2. SODs serve as the initial defense by converting superoxide radicals into hydrogen peroxide, which is metabolized to water by the enzymes CAT and GPx [38]. The mRNA levels of *Sod1* and *Gpx1* were increased in the 10-G group, being significant only for the latter. This is contrary to what has been reported for liver tissue, where ginger supplementation significantly upregulated hepatic antioxidant enzymes involved in ROS elimination, including SOD 1/2 and nuclear factor erythroid 2-related factor 2 (Nrf-2), but not GPx [39]. GPxs are crucial in defending against oxidative damage by managing intracellular hydrogen peroxide and reducing organic peroxides, including fatty acid hydroperoxides, using glutathione (GSH) as a reducing agent to convert hydrogen peroxide into water and oxygen [40,41]. The protein levels of GPx and CAT were significantly increased in the 10-G group. The CAT enzyme neutralizes two hydrogen peroxide molecules into water and oxygen, preventing the production of hydroxyl radicals [38]. Although it was not addressed in this study, some studies have demonstrated that gingerols upregulated the activity of antioxidant enzymes, including SODs and GPxs [42,43,44]. The results of a meta-analysis published in 2021 showed that ginger intake significantly increased GPx activity and total antioxidant capacity and significantly decreased MDA levels compared to control groups [44]. Thus, the antioxidant properties of 10-gingerol could play a key role in modulating chronic inflammation by inducing the production of endogenous antioxidative enzymes that protect cells from oxidative damage caused by ROS and from the inflammatory processes that these can trigger in the body. This makes antioxidant compounds like 10-gingerol from ginger promising in the treatment and prevention of oxidative stress and inflammation-related diseases.

Adipose tissue exerts an endocrine function by producing a variety of proteins, e.g., adipokines, which are involved in regulating adipogenesis, energy metabolism, and the immune response [45]. However, when there is an excess of adipose tissue and adipocyte hypertrophy, it leads to an alteration in adipokine production, which partly contributes to the metabolic dysfunction of the tissue and the development of the low-grade chronic inflammation characteristic of obesity [3,8,9].

Several authors have suggested that ginger compounds could play an important role in restoring the inflammatory state associated with obesity by regulating the production of adipokines. In vivo models of obese mice have shown that supplementation with 40 and 80 mg/kg of an ethanolic ginger extract increased the relative expression levels of adiponectin in adipose tissue [46], while treatment with 75 mg/kg of 6-gingerol decreased TNFα protein expression in adipose tissue [47]. Similarly, Suk and collaborators in 2017 reported that treatment with 50 mg/kg of gingerenone-A increased the relative expression levels of *Acrp30* and reduced the levels of *Tnf* in adipose tissue [22]. Furthermore, Gunawan and collaborators showed that in obese and insulin-resistant rats, treatment with 200 mg/kg/day of 6-gingerol increased adiponectin concentrations and decreased TNFα levels in adipose tissue [48].

Moreover, in vitro studies with ginger phenols such as 6-gingerol [46] and 6-shogaol [49] have been shown to regulate adipokine production in 3T3-L1 adipocytes. However, these studies have not considered stimulation for an inflammatory environment. On the other hand, Jolad et al. found that organic ginger extracts effectively inhibited LPS-induced PGE2 production in HL-60 cells. These extracts, rich in gingerols and shogaols as identified by HPLC, contained 5–8% 10-gingerol, corresponding to a concentration of 0.53 to 4 μg/mL. Although this is much lower compared to the dose administered in our study, it is crucial to consider that these extracts contain gingerols and other natural compounds that act synergistically to achieve a therapeutic effect. Our dose of 15 μg/mL is comparable to that of 6-gingerol in an extract of fresh yellow ginger, where 6-gingerol, the primary gingerol, reached an estimated concentration of 16.9 μg/mL [50]. Thus, our dose is within the range used for other gingerols in ginger extracts, demonstrating the anti-inflammatory effectiveness of this pure compound.

In our results, treatment with 10-gingerol in 3T3-L1 adipocytes showed an anti-inflammatory effect by increasing the production of adiponectin and decreasing TNFα levels following acute LPS stimulation. Isa and collaborators showed that stimulation with 25 µM of 6-shogaol increased adiponectin expression and decreased TNFα expression levels in 3T3-L1 adipocytes [49]. Additionally, other studies have demonstrated that treatment with 25 and 50 µg/mL of an ethanolic ginger extract increased the relative expression levels of adiponectin in 3T3-L1 adipocytes [51], while in co-cultures of 3T3-L1 adipocytes with RAW264.7 macrophages, treatment with 10 µM of gingerenone-A decreased the mRNA expression of *Tnf* [22]. 

Adiponectin is a hormone produced exclusively by adipocytes, exhibiting anti-inflammatory and insulin-sensitizing properties [45]. Through the activation of AMP-activated protein kinase (AMPK) and p38 mitogen-activated protein kinases (p38MAPK), adiponectin promotes the insulin-responsive glucose transporter type 4 (GLUT4) translocation to the membrane, inhibits lipogenic pathways, enhances fatty acid oxidation in white adipose tissue, and inhibits the expression of pro-inflammatory genes, including TNFα, by blocking the IKB kinase (IKK)-NF-κB signaling pathway [52,53]. This improves insulin resistance and contributes to reducing chronic inflammation associated with obesity [54,55]. While TNFα is a cytokine produced by macrophages, adipocytes, and preadipocytes, playing a role in cross-talk between these cells, thereby promoting synergistic inflammation development [45,56]. In addition to its inflammatory effect, TNFα has been shown to downregulate peroxisome proliferator-activated receptor gamma (PPARγ) expression, thereby reducing adiponectin production and the expression of enzymes involved in de novo fatty acid synthesis, altering fat cell metabolism [52]. These findings suggest the potential anti-inflammatory effect of 10-gingerol in LPS-stimulated fat cells.

Furthermore, stimulation with 10-gingerol also decreased the levels of resistin in the supernatant. These findings represent the first evidence of ginger compounds affecting resistin production in 3T3-L1 adipocytes. Resistin is an adipokine secreted by adipocytes and preadipocytes, and its expression is crucial for lipid accumulation in adipocytes [52,57]. However, overproduction of resistin due to increased adipose tissue reduces adiponectin signaling, leading to insulin resistance and increased production of inflammatory cytokines [7,52]. Therefore, the reduction in resistin levels following stimulation with 10-gingerol complements the anti-inflammatory effect that this phenol could exert in an obese state. 

Regarding leptin, our study revealed an increase in the concentrations of this adipokine in the supernatant following stimulation with 10-gingerol. Leptin is a peptide hormone secreted mainly from adipose tissue, which plays an integral role in the regulation of body weight and energy expenditure [58]. Studies on leptin have elucidated the complex interplay connecting nutrition, metabolism, reproduction, immune functions, and inflammation [59,60]. The leptin receptor is widely expressed on the surface of various immune cells, including peripheral cells like monocytes/macrophages, as well as T and B cells. Multiple pathways are involved in its signaling, with the Janus kinase (JAK) signal transducer and activator of transcription (JAK-STAT) pathway being one of the primary cascades triggered by leptin to induce immune cell activation [59]. On the other hand, leptin’s effects on lipid metabolism regulation are similar to adiponectin, acting through AMPK phosphorylation to promote lipolysis and inhibit lipogenesis in muscles, liver, and adipose tissue [52,61].

Notably, obesity is associated with hyperleptinemia and leptin resistance, which compromises its effect on satiety and metabolic functions, making the reduction in leptin levels beneficial [62,63]. In models of mice with high-fat-diet-induced obesity, a decrease in leptin concentrations and expression levels has been reported following treatment with 6-gingerol [63] or with freeze-dried fresh ginger [62]. Our study was conducted using adipocyte cultures where neither hyperleptinemia nor leptin resistance were present; hence, our results cannot be directly comparable to previous findings on other phenols. It should also be considered that, in an organism, leptin synthesis is primarily regulated by food intake and eating-related hormones. This regulation depends on energy status, sex hormones (being inhibited by testosterone and increased by ovarian steroids), a wide range of inflammation mediators (being promoted or inhibited by pro-inflammatory cytokines), and the inflammatory context (depending on whether their action is acute or chronic) [59,64].

On the other hand, IL-6 is an adipokine primarily produced by macrophages, followed by fibroblasts, and to a lesser extent by adipocytes [56]. IL-6 plays a crucial role in maintaining the inflammatory state by promoting macrophage differentiation towards a pro-inflammatory (M1) profile. Additionally, it reduces adiponectin expression and secretion and promotes free fatty acid release in adipose tissue [45,56]. In models of obesity supplemented with 6-gingerol, a decrease in IL-6 concentrations in serum and adipose tissue has been evidenced [48], as well as in the expression at the mRNA level [63] and the protein level [47] of this adipokine. Our results did not show significant differences in the concentrations of this adipokine between the positive control group and the 10-G group stimulated with LPS. In fact, in adipose tissue cultures, adipocytes produce less than 10% IL-6 [65]. Compared with other studies, in BV2 microglial cell cultures activated with LPS and stimulated with 10-gingerol, a decrease in IL-6 concentrations at both the protein and mRNA levels has been observed [66,67]. Some of the reasons that could explain these discrepancies include the higher dose and longer duration of LPS stimulation used in those studies compared to ours. 

Although this study did not investigate the potential mechanisms underlying the observed effects, these may be related to those described for other homologs, such as 6-gingerol and 6-shogaol. It has been proposed that these compounds might enhance antioxidant defense mechanisms through the induction of Nrf2 and HO-1, which are regulated by the p38 MAPK and phosphatidylinositol 3-kinase (PI3K)/protein kinase B (AKT) pathways [68]. Additionally, their anti-inflammatory effects could be associated with the inhibition of NF-κB activation, as well as the phosphorylation of the extracellular signal-regulated kinase (ERK) 1/2 and p38 MAPKs [69]. 

Our study has certain limitations. Firstly, this study investigated the effects of 10-gingerol in a single cell line; consequently, we were unable to assess whether using co-cultures of 3T3-L1 adipocytes with RAW264.7 macrophages may influence the observed outcomes. Secondly, we did not evaluate ROS formation or antioxidant enzymatic activities, which could have been used to confirm the functionality of CAT, SOD, and GPx. Thirdly, the results were not compared with a standard molecule with known antioxidant capacity. Finally, extending the exposure time to LPSs could be useful to improve the inflammation environment. Therefore, future research will be needed to assess these limitations to better understand its antioxidant and anti-inflammatory effects. 

## 5. Conclusions

In conclusion, we showed that 10-gingerol has a low capacity to scavenge free radicals. Moreover, 10-gingerol presents antioxidant potential by increasing CAT and GPx abundance and ameliorates LPS-induced inflammation through increasing adiponectin and decreasing TNFα and resisitin levels in 3T3-L1 adipocytes. This study shows that 10-gingerol could have an antioxidant and anti-inflammatory effect on adipocytes that could contribute to improving metabolism by increasing endogenous antioxidants and modulating adipokine levels. Transitioning the study to an in vivo obesity model would help confirm these properties of 10-gingerol and could clarify its potential implications for combating obesity-related oxidative stress and inflammation.

## Data Availability

The original data presented in the study are openly available in the zenodo repository at https://doi.org/10.5281/zenodo.12702098. (accessed on 9 July 2024).

## Supplementary Materials

The following supporting information can be downloaded at https://www.mdpi.com/article/10.3390/antiox13091093/s1, Table S1: Dose-response curve data in 3T3-L1 adipocytes.

## References

[1] Caballero B. (2019). Humans against Obesity: Who Will Win?. Adv. Nutr..

[2] Obesity and Overweight. https://www.who.int/news-room/fact-sheets/detail/obesity-and-overweight.

[3] González-Muniesa P., Mártinez-González M.-A., Hu F.B., Després J.-P., Matsuzawa Y., Loos R.J.F., Moreno L.A., Bray G.A., Martinez J.A. (2017). Obesity. Nat. Rev. Dis. Primers.

[4] Kawai T., Autieri M.V., Scalia R. (2021). Adipose Tissue Inflammation and Metabolic Dysfunction in Obesity. Am. J. Physiol.-Cell Physiol..

[5] Longo M., Zatterale F., Naderi J., Parrillo L., Formisano P., Raciti G.A., Beguinot F., Miele C. (2019). Adipose Tissue Dysfunction as Determinant of Obesity-Associated Metabolic Complications. Int. J. Mol. Sci..

[6] Li H., Ren J., Li Y., Wu Q., Wei J. (2023). Oxidative Stress: The Nexus of Obesity and Cognitive Dysfunction in Diabetes. Front. Endocrinol..

[7] Luo L., Liu M. (2016). Adipose Tissue in Control of Metabolism. J. Endocrinol..

[8] Andersen C.J., Murphy K.E., Fernandez M.L. (2016). Impact of Obesity and Metabolic Syndrome on Immunity. Adv. Nutr..

[9] Ghaben A.L., Scherer P.E. (2019). Adipogenesis and Metabolic Health. Nat. Rev. Mol. Cell Biol..

[10] De Mello A.H., Costa A.B., Engel J.D.G., Rezin G.T. (2018). Mitochondrial Dysfunction in Obesity. Life Sci..

[11] Jakubiak G.K., Osadnik K., Lejawa M., Kasperczyk S., Osadnik T., Pawlas N. (2021). Oxidative Stress in Association with Metabolic Health and Obesity in Young Adults. Oxid. Med. Cell Longev..

[12] Foncea R., Carvajal C., Almarza C., Leighton F. (2000). Endothelial Cell Oxidative Stress and Signal Transduction. Biol. Res..

[13] Bowie A., O’Neill L.A.J. (2000). Oxidative Stress and Nuclear Factor-κB Activation. Biochem. Pharmacol..

[14] Furukawa S., Fujita T., Shimabukuro M., Iwaki M., Yamada Y., Nakajima Y., Nakayama O., Makishima M., Matsuda M., Shimomura I. (2017). Increased Oxidative Stress in Obesity and Its Impact on Metabolic Syndrome. J. Clin. Investig..

[15] Masenga S.K., Kabwe L.S., Chakulya M., Kirabo A. (2023). Mechanisms of Oxidative Stress in Metabolic Syndrome. Int. J. Mol. Sci..

[16] Korac B., Kalezic A., Pekovic-Vaughan V., Korac A., Jankovic A. (2021). Redox Changes in Obesity, Metabolic Syndrome, and Diabetes. Redox Biol..

[17] Antioxidants and Phytochemicals—A Scoping Review for Nordic Nutrition Recommendations 2023|Food & Nutrition Research. https://foodandnutritionresearch.net/index.php/fnr/article/view/10324.

[18] Nani A., Murtaza B., Sayed Khan A., Khan N.A., Hichami A. (2021). Antioxidant and Anti-Inflammatory Potential of Polyphenols Contained in Mediterranean Diet in Obesity: Molecular Mechanisms. Molecules.

[19] Tzeng T.-F., Liu I.-M. (2013). 6-Gingerol Prevents Adipogenesis and the Accumulation of Cytoplasmic Lipid Droplets in 3T3-L1 Cells. Phytomedicine.

[20] Foods|Free Full-Text|Bioactive Compounds and Bioactivities of Ginger (*Zingiber officinale Roscoe*). https://www.mdpi.com/2304-8158/8/6/185.

[21] Preciado-Ortiz M.E., Martinez-Lopez E., Rodriguez-Echevarría R., Perez-Robles M., Gembe-Olivarez G., Rivera-Valdés J.J. (2023). 10-Gingerol, a Novel Ginger Compound, Exhibits Antiadipogenic Effects without Compromising Cell Viability in 3T3-L1 Cells. Biomed. Rep..

[22] Suk S., Kwon G.T., Lee E., Jang W.J., Yang H., Kim J.H., Thimmegowda N.R., Chung M.-Y., Kwon J.Y., Yang S. (2017). Gingerenone A, a Polyphenol Present in Ginger, Suppresses Obesity and Adipose Tissue Inflammation in High-Fat Diet-Fed Mice. Mol. Nutr. Food Res..

[23] Liu Y., Liu J., Zhang Y. (2019). Research Progress on Chemical Constituents of *Zingiber officinale* Roscoe. BioMed Res. Int..

[24] Baliga M.S., Haniadka R., Pereira M.M., D’Souza J.J., Pallaty P.L., Bhat H.P., Popuri S. (2011). Update on the Chemopreventive Effects of Ginger and Its Phytochemicals. Crit. Rev. Food Sci. Nutr..

[25] Yücel Ç., Karatoprak G.Ş., Açıkara Ö.B., Akkol E.K., Barak T.H., Sobarzo-Sánchez E., Aschner M., Shirooie S. (2022). Immunomodulatory and Anti-Inflammatory Therapeutic Potential of Gingerols and Their Nanoformulations. Front. Pharmacol..

[26] Zick S.M., Djuric Z., Ruffin M.T., Litzinger A.J., Normolle D.P., Alrawi S., Feng M.R., Brenner D.E. (2008). Pharmacokinetics of 6-Gingerol, 8-Gingerol, 10-Gingerol, and 6-Shogaol and Conjugate Metabolites in Healthy Human Subjects. Cancer Epidemiol. Biomark. Prev..

[27] Fernández-Rojas B., Gómez-Sierra T., Medina-Campos O.N., Hernández-Juárez J., Hernández-Cruz P.A., Gallegos-Velasco I.B., Pérez-Cervera Y., Pedraza-Chaverri J. (2023). Antioxidant Activity of Glucosamine and Its Effects on ROS Production, Nrf2, and O-GlcNAc Expression in HMEC-1 Cells. Curr. Res. Toxicol..

[28] Ignacio-Mejía I., Contreras-García I.J., Mendoza-Torreblanca J.G., Medina-Campos O.N., Pedraza-Chaverri J., García-Cruz M.E., Romo-Mancillas A., Gómez-Manzo S., Bandala C., Sánchez-Mendoza M.E. (2023). Evaluation of the Antioxidant Activity of Levetiracetam in a Temporal Lobe Epilepsy Model. Biomedicines.

[29] Jumina J., Siswanta D., Zulkarnain A.K., Triono S., Priatmoko P., Yuanita E., Fatmasari N., Nursalim I. (2019). Development of C-Arylcalix [4]Resorcinarenes and C-Arylcalix [4]Pyrogallolarenes as Antioxidant and UV-B Protector. Indones. J. Chem..

[30] Alliin, a Garlic (*Allium sativum*) Compound, Prevents LPS-Induced Inflammation in 3T3-L1 Adipocytes—Quintero-Fabián—2013—Mediators of Inflammation—Wiley Online Library. https://onlinelibrary.wiley.com/doi/10.1155/2013/381815.

[31] Livak K.J., Schmittgen T.D. (2001). Analysis of Relative Gene Expression Data Using Real-Time Quantitative PCR and the 2(-Delta Delta C(T)) Method. Methods.

[32] Yap V.L., Tan L.F., Rajagopal M., Wiart C., Selvaraja M., Leong M.Y., Tan P.L. (2023). Evaluation of Phytochemicals and Antioxidant Potential of a New Polyherbal Formulation TC-16: Additive, Synergistic or Antagonistic?. BMC Complement. Med. Ther..

[33] Ninfali P., Mea G., Giorgini S., Rocchi M., Bacchiocca M. (2005). Antioxidant Capacity of Vegetables, Spices and Dressings Relevant to Nutrition. Br. J. Nutr..

[34] Ghasemzadeh A., Jaafar H.Z.E., Rahmat A. (2010). Antioxidant Activities, Total Phenolics and Flavonoids Content in Two Varieties of Malaysia Young Ginger (*Zingiber officinale* Roscoe). Molecules.

[35] Liew S.S., Ho W.Y., Yeap S.K., Sharifudin S.A.B. (2018). Phytochemical Composition and in Vitro Antioxidant Activities of Citrus Sinensis Peel Extracts. PeerJ.

[36] Tinello F., Zannoni S., Lante A. (2020). Antioxidant Properties of Soybean Oil Supplemented with Ginger and Turmeric Powders. Appl. Sci..

[37] Miliauskas G., Venskutonis P.R., van Beek T.A. (2004). Screening of Radical Scavenging Activity of Some Medicinal and Aromatic Plant Extracts. Food Chem..

[38] Ayustaningwarno F., Anjani G., Ayu A.M., Fogliano V. (2024). A Critical Review of Ginger’s (*Zingiber officinale*) Antioxidant, Anti-Inflammatory, and Immunomodulatory Activities. Front. Nutr..

[39] Seo S.H., Fang F., Kang I. (2021). Ginger (*Zingiber officinale)* Attenuates Obesity and Adipose Tissue Remodeling in High-Fat Diet-Fed C57BL/6 Mice. Int. J. Environ. Res. Public Health.

[40] Limón-Pacheco J., Gonsebatt M.E. (2009). The Role of Antioxidants and Antioxidant-Related Enzymes in Protective Responses to Environmentally Induced Oxidative Stress. Mutat. Res..

[41] Jena A.B., Samal R.R., Bhol N.K., Duttaroy A.K. (2023). Cellular Red-Ox System in Health and Disease: The Latest Update. Biomed. Pharmacother..

[42] Integrated Chromatographic Approach for the Discovery of Gingerol Antioxidants from Dracocephalum Heterophyllum and Their Potential Targets—Analytical Methods (RSC Publishing). https://pubs.rsc.org/en/content/articlelanding/2022/ay/d2ay01282k.

[43] Abdurrahim A.E., Mazurak V.C., Chen L. (2023). Gingerols Synergize with Anthocyanins to Induce Antioxidant Activity in Vitro. Front. Nutr..

[44] Morvaridzadeh M., Sadeghi E., Agah S., Fazelian S., Rahimlou M., Kern F.G., Heshmati S., Omidi A., Persad E., Heshmati J. (2021). Effect of Ginger (*Zingiber officinale*) Supplementation on Oxidative Stress Parameters: A Systematic Review and Meta-Analysis. J. Food Biochem..

[45] Scheja L., Heeren J. (2019). The Endocrine Function of Adipose Tissues in Health and Cardiometabolic Disease. Nat. Rev. Endocrinol..

[46] Kim B., Kim H.-J., Cha Y.-S. (2021). The Protective Effects of Steamed Ginger on Adipogenesis in 3T3-L1 Cells and Adiposity in Diet-Induced Obese Mice. Nutr. Res. Pract..

[47] Brahma Naidu P., Uddandrao V.V.S., Ravindar Naik R., Suresh P., Meriga B., Begum M.S., Pandiyan R., Saravanan G. (2016). Ameliorative Potential of Gingerol: Promising Modulation of Inflammatory Factors and Lipid Marker Enzymes Expressions in HFD Induced Obesity in Rats. Mol. Cell. Endocrinol..

[48] Gunawan S., Munika E., Wulandari E.T., Ferdinal F., Purwaningsih E.H., Wuyung P.E., Louisa M., Soetikno V. (2023). 6-Gingerol Ameliorates Weight Gain and Insulin Resistance in Metabolic Syndrome Rats by Regulating Adipocytokines. Saudi Pharm. J..

[49] Isa Y., Miyakawa Y., Yanagisawa M., Goto T., Kang M.-S., Kawada T., Morimitsu Y., Kubota K., Tsuda T. (2008). 6-Shogaol and 6-Gingerol, the Pungent of Ginger, Inhibit TNF-α Mediated Downregulation of Adiponectin Expression via Different Mechanisms in 3T3-L1 Adipocytes. Biochem. Biophys. Res. Commun..

[50] Jolad S.D., Lantz R.C., Chen G.J., Bates R.B., Timmermann B.N. (2005). Commercially Processed Dry Ginger (*Zingiber officinale*): Composition and Effects on LPS-Stimulated PGE2 Production. Phytochemistry.

[51] Li H., Rafie A.R., Hamama A., Siddiqui R.A. (2023). Immature Ginger Reduces Triglyceride Accumulation by Downregulating Acyl CoA Carboxylase and Phosphoenolpyruvate Carboxykinase-1 Genes in 3T3-L1 Adipocytes. Food Nutr. Res..

[52] Lago F., Gómez R., Gómez-Reino J.J., Dieguez C., Gualillo O. (2009). Adipokines as Novel Modulators of Lipid Metabolism. Trends Biochem. Sci..

[53] Khoramipour K., Chamari K., Hekmatikar A.A., Ziyaiyan A., Taherkhani S., Elguindy N.M., Bragazzi N.L. (2021). Adiponectin: Structure, Physiological Functions, Role in Diseases, and Effects of Nutrition. Nutrients.

[54] Garcia D., Shaw R.J. (2017). AMPK: Mechanisms of Cellular Energy Sensing and Restoration of Metabolic Balance. Mol. Cell.

[55] Lee G.-H., Peng C., Jeong S.-Y., Park S.-A., Lee H.-Y., Hoang T.-H., Kim J., Chae H.-J. (2021). Ginger Extract Controls mTOR-SREBP1-ER Stress-Mitochondria Dysfunction through AMPK Activation in Obesity Model. J. Funct. Foods.

[56] Makki K., Froguel P., Wolowczuk I. (2013). Adipose Tissue in Obesity-Related Inflammation and Insulin Resistance: Cells, Cytokines, and Chemokines. Int. Sch. Res. Not..

[57] Ikeda Y., Tsuchiya H., Hama S., Kajimoto K., Kogure K. (2013). Resistin Affects Lipid Metabolism during Adipocyte Maturation of 3T3-L1 Cells. FEBS J..

[58] Zhang Y., Proenca R., Maffei M., Barone M., Leopold L., Friedman J.M. (1994). Positional Cloning of the Mouse Obese Gene and Its Human Homologue. Nature.

[59] Pérez-Pérez A., Sánchez-Jiménez F., Vilariño-García T., Sánchez-Margalet V. (2020). Role of Leptin in Inflammation and Vice Versa. Int. J. Mol. Sci..

[60] Iikuni N., Lam Q.L.K., Lu L., Matarese G., La Cava A. (2008). Leptin and Inflammation. Curr. Immunol. Rev..

[61] Zeng W., Pirzgalska R.M., Pereira M.M.A., Kubasova N., Barateiro A., Seixas E., Lu Y.-H., Kozlova A., Voss H., Martins G.G. (2015). Sympathetic Neuro-Adipose Connections Mediate Leptin-Driven Lipolysis. Cell.

[62] Sayed S., Ahmed M., El-Shehawi A., Alkafafy M., Al-Otaibi S., El-Sawy H., Farouk S., El-Shazly S. (2020). Ginger Water Reduces Body Weight Gain and Improves Energy Expenditure in Rats. Foods.

[63] Hong K.H., Um M.Y., Ahn J., Ha T.Y. (2023). 6-Gingerol Ameliorates Adiposity and Inflammation in Adipose Tissue in High Fat Diet-Induced Obese Mice: Association with Regulating of Adipokines. Nutrients.

[64] Gualillo O., Eiras S., Lago F., Diéguez C., Casanueva F.F. (2000). Elevated Serum Leptin Concentrations Induced by Experimental Acute Inflammation. Life Sci..

[65] Coppack S.W. (2001). Pro-Inflammatory Cytokines and Adipose Tissue. Proc. Nutr. Soc..

[66] Dugasani S., Pichika M.R., Nadarajah V.D., Balijepalli M.K., Tandra S., Korlakunta J.N. (2010). Comparative Antioxidant and Anti-Inflammatory Effects of [6]-Gingerol, [8]-Gingerol, [10]-Gingerol and [6]-Shogaol. J. Ethnopharmacol..

[67] Ho S.-C., Chang K.-S., Lin C.-C. (2013). Anti-Neuroinflammatory Capacity of Fresh Ginger Is Attributed Mainly to 10-Gingerol. Food Chem..

[68] Bak M.-J., Ok S., Jun M., Jeong W.-S. (2012). 6-Shogaol-Rich Extract from Ginger up-Regulates the Antioxidant Defense Systems in Cells and Mice. Molecules.

[69] Choi Y.Y., Kim M.H., Hong J., Kim S.-H., Yang W.M. (2013). Dried Ginger (*Zingiber officinalis*) Inhibits Inflammation in a Lipopolysaccharide-Induced Mouse Model. Evid.-Based Complement. Altern. Med..

