# Supplementary material for: 10-Gingerol Increases Antioxidant Enzymes and Attenuates Lipopolysaccharide-Induced Inflammation by Modulating Adipokines in 3T3-L1 Adipocytes
Source: Antioxidants (Basel). 2024 Sep 7;13(9):1093. doi: 10.3390/antiox13091093 (PMC11429246; doi:10.3390/antiox13091093)
Supplement: Supplementary file 1 [file antioxidants-13-01093-s001.zip › antioxidants-3129072-supplementary.pdf]

Supplemental material Table S1

Dose-response curve data in 3T3-L1 adipocytes.

% cell viability by MTT assay				
**Vehicle**	**Control**	**15 µg/ml**	**25 µg/ml**	**35 µg/ml**
100.85	100	111.14	83.06	70.79
101.61	95.56	117.65	86.86	69.86
97.29	105	114.55	81.65	70.29

% lipid accumulation by oil red O staining				
**Vehicle**	**Control**	**15 µg/ml**	**25 µg/ml**	**35 µg/ml**
113	108.22	61.71	55.24	44.99
89.19	97.46	59.50	55.24	54.27
101.09	94.30	47.57	54.97	44.90
